# Effects of Milling and Cooking Processes on the Deoxynivalenol Content in Wheat

**DOI:** 10.3390/ijms9112127

**Published:** 2008-11-05

**Authors:** Masayo Kushiro

**Affiliations:** National Food Research Institute, National Agriculture and Food Research Organization / 2-1-12 Kannondai, Tsukuba 305-8642, Japan. E-Mail: kushirom@affrc.go.jp; Tel. +81-29-838-8069; Fax: +81-29-838-7996

**Keywords:** Deoxynivalenol, DON, wheat, milling, cooking

## Abstract

Deoxynivalenol (DON, vomitoxin) is a natural-occuring mycotoxin mainly produced by *Fusarium graminearum*, a food-borne fungi widely distributed in crops and it is one of the most important mycotoxins in wheat and wheat-based foods and feeds. DON affects animal and human health causing diarrhea, vomiting, gastro-intestinal inflammation, and immunomodulation. Since the rate of the occurrence of DON in wheat is high, effective procedures to remove or eliminate DON from food products is essential to minimize exposures in those who consume large amounts of wheat. Cleaning prior to milling reduced to some extent the concentration of DON in final products. Since DON is distributed throughout the kernels, with higher content in the outer skin, milling is also effective in reducing the DON levels of wheat-based foods if bran and shorts are removed before thermal cooking. DON is water-soluble and cooking with larger amounts of water lowers DON content in products such as spaghetti and noodles. During baking or heating, DON is partially degraded to DON-related chemicals, whose toxicological effects are not studied well. This paper reviews the researches on the effects of milling and cooking on the DON level and discusses the perspectives of further studies.

## 1. Introduction

**Figure 1.**
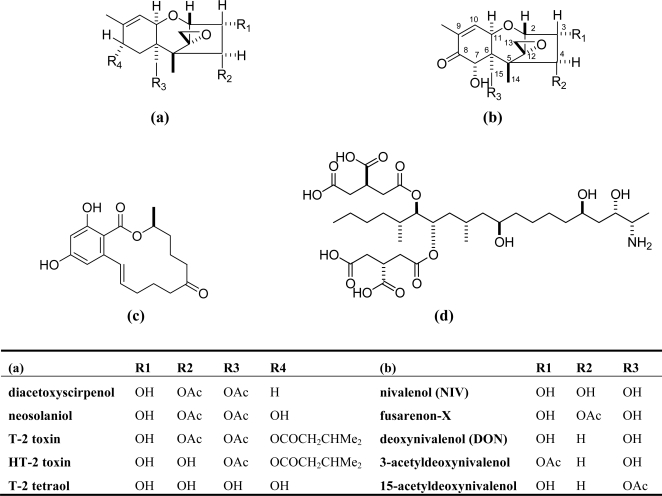
Chemical structures of major *Fusarium* mycotoxins. (a) Type A trichothecenes (b) Type B trichothecenes (c) zearalenone (ZON) (d) fumonisin B1.

The mycotoxin deoxynivalenol (DON) or vomitoxin is a major metabolite produced by *Fusarium graminearum* (Teleomorph = *Gibberella zeae*), one of the most common fungi associated world-wide with grains in the field [1]. Besides *F. graminearum*, *F. culmorum* is a major plant pathogen, both causing *Fusarium* head blight (FHB) or scab in wheat and barley, and *Gibberella* ear rot in maize [2]. Like other *Fusarium* toxins such as zearalenone (ZON) and fumonisins, climatic conditions during the growth have the major influence on these plant diseases. The incidence of FHB is most affected by the rainfall at the timing of flowering, however, DON content is not always correlated with the severity of this symptom [3]. Chemically, DON is a member of the type B trichothecenes or 8-ketotrichothecenes, which consists of hundreds of compounds [4]. DON is often found to be co-exist with another two *Fusarium* mycotoxins nivalenol (NIV, a type B trichothecene) and ZON (not a trichothecene) in scabby grains (Figure 1 **(b)**, **(c)**). So far, regulatory values are set only for DON in many countries because of its worldwide occurrence including North America. The higher occurrence of NIV compared to DON was occasionally reported in Northern Europe and Asia [5, 6].

DON was found to be the compound responsible for emesis and feed refusal in swine [7] and it has been shown to be a strong emetic agent in swine, dogs, and ducklings [8, 9]. They are all non-volatile, low-molecular-weight sesquiterpene epoxides, sharing a tricyclic nucleus named trichothecene and usually contain an epoxide at C-12 and C-13, which are essential for toxicity [10]. Most trichothecenes have a C-9,10 double bond, which is also considered to be important for their toxicity [11] (Figure 1). So far, toxicological assessment of type B trichothecenes has for the most part centered on DON, the most prevalent compound in wheat field all over the world (Table 1). DON have been reported to exhibit various toxicological effects such as reduction of growth and feed consumption (anorexia) at low concentrations in the diet whereas it induces vomiting (emesis) at higher acute doses in animals [12]. The ingestion of DON in mice mimics the common human glomerulonephritis, IgA nephropathy [13]. At the molecular level, DON is a noncompetitive potent protein synthesis inhibitor that can significantly alter humoral immunity, cell-mediated immunity in a variety of eukaryotic cells including experimental animal models [14, 15]. Although there are numerous reports on the toxicological effects of DON, it is not considered to be a mutagen nor carcinogen. Various reviews on the toxicity of DON can be found in the literature or website [12, 16–19, 22] (Table 1).

DON contamination of wheat is a great concern to animal and human health. Prevention of *Fusarium* toxins including DON has been intensively studied by plant physiologists and plant pathologists all over the world because genera *Fusarium* are typical field fungi infecting plants on the field. They infect plant hosts and cause disease in the field, which result in not only reduction of harvest but also accumulation of several mycotoxins [24]. While there is a vast knowledge exists for the occurrence and prevention of *Fusarium* fungi on the field, the knowledge on the retention of DON after harvest and during processing is limited. On the other hand, approximately 600 million tons of wheat are produced per year worldwide and most of them are converted to wheat flour for human consumption, which is not the case of corn, of which the majority is used directly for animal feed. Much of the wheat flour will be then processed into various foods such as bread, pasta, noodles, and cakes. Therefore, the study on the retention of DON during primary processing (milling) and secondary processing (cooking) is important for the risk assessment and management for the people who live on the wheat-based food products. This article reviews the results of studies on the retention of DON during milling and cooking and discusses the directions for further studies.

## 2. Effects of primary processing; cleaning, scouring, density segregation and milling on the retention of DON

Harvested wheat kernels are converted to wheat flour for human consumption. The primary processing consists of selection and milling. Selection or separation of visually uninfected kernels is done by the properties of kernels, such as shape, size, relative density and resistance to air. The milling process for wheat is a dry milling after tempering (adding water to dry grain and allowing it to rest for a while). Many experiments are performed using various kinds of wheat kernels of naturally FHB-infected wheat or test samples artificially infected by *F. graminearum* (*G. zeae*). These kernels are contaminated with various level of DON from low-level (<0.1 mg/kg) to high-level (>5.0 mg/kg).

### 2.1. Effects of selection processes prior to milling on the removal of DON

Various equipment with either a single function or multiple functions of sorting, sieving and scouring are available and they are tested for the removal efficiency of DON containing kernels from the mixture of visually uninfected kernels and scabby ones.

DON could be removed on each process prior to milling to some extent. Scott *et al.* [19] showed that cleaning was effective because higher concentrations of DON were found in dockage. However, only a slight reduction was expected since the dockage accounts for only 3.2% of the weight of the parts where DON present. Generally cleaning was partially effective on removal of DON in various variety of wheat [20–24, 26]. Nowicki *et al*. [24] reported that scouring was effective because the toxin was unevenly distributed in the surface of kernels, and DON-producing fungus was also removed from the surface. Since scabby kernels have lower relative density, some studies revealed that the selection by gravity was more effective to remove heavily infected kernels with high-content of DON from visually uninfected kernels, especially in case of highly DON-contaminated wheat sample [25, 27] (Table 2). Selection of *Fusarium*-infected kernels by spectrometric analysis such as mid-infrared spectroscopy or fluorescence analysis is also remained to be studied [34]. In all cases, the problem situates on the point that FHB level is not always correlated with the content of DON in the kernels and occasionally some level of DON is contained in visually uninfected kernels [3].

### 2.2. Effects of dry milling and DON distribution for each milling fraction

Most of the wheat harvested is processed in industrial scale mills for human consumption. Numbers of data are obtained by laboratory scale test mills. Test mills contain basic components for dry milling without purifier. Results have been reported from milling tests using wheat cultivars from Canada, USA, Korea, Japan since 1980’s, and recently from Italy, Czech Republic, and Switzerland (Table 3).

To some extent milling reduces DON levels in straight-grade flour by fractionation [25, 32, 38]. DON was found in the highest amounts in fractions of the commodity that are less likely to be used for food production (germ and bran fractions), but the toxin was distributed throughout the milling fractions basically independently of wheat variety [25–29, 35, 42–46, 48]. Cultivar dependency of kernel site where the fungus can invade, and cultivar specificity of reduction ratio of DON during milling were found in some cultivars of red spring wheat [30, 38]. It is reported that a longer tempering time is a possible cause of increase of total DON content after milling, because of propagation of DON-producing fungi during tempering [35].

## 3. Effects of secondary processing; cooking with heating on the retention of DON

Numerous studies have documented that DON is heat-stable. DON is very stable during baking at the temperature of 170 °C –350 °C, with no reduction of DON concentration after 30 min at 170 °C [25, 37, 48, 50] (Table 4). DON levels are reduced in cooked pasta and noodles because of leaching into the cooking water [30, 44, 53] since DON is water-soluble, while no reduction was observed in frying in oil [55]. Some evidence indicates that DON levels may be reduced during the processing in basic condition such as boiling of Chinese noodles (containing Kansui: a commercial preparation of carbonate and phosphate salts of potassium and sodium) [30].

Extrusion cooking is one of the fastest growing food-processing operations in recent years due to several advantages over traditional methods. Reductions of 100, 95 and 83% for fumonisins, aflatoxins and ZON, respectively, have been reported during extrusion cooking of corn, while lower reductions were observed for DON, ochratoxin A and moniliformin, where maximum reductions did not exceed 55, 40 and 30%, respectively [58]. Accerbi *et al.* reported 62% reduction of DON in wheat during extrusion [52] in the presence of sodium bisulfate. Generally, no reduction of DON content by simple extrusion of wheat flour was observed [59].

Recently, an application of a new technology, superheated steam, for the reduction of DON in naturally *Fusarium*-infected wheat was reported by Cenkowski *et al.* [60]. A 48% reduction of DON was achieved with a steam temperature of 185°C and a processing time of 6 min.

**Figure 2.**
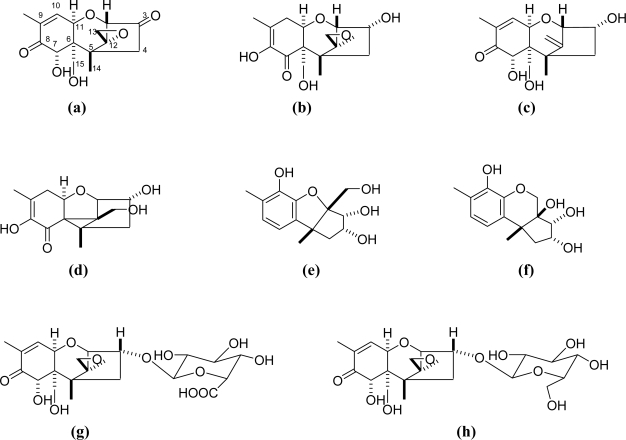
Chemical structures of derivaives of DON. **(a)** 3-keto DON **(b)** isoDON **(c)** de-epoxy DON **(d)** norDON A **(e)** norDON B **(f)** norDON C **(g)** DON-3-glucuronide **(h)** DON-3-glucoside (DON-3G).

As for baking, some reports describe that heating reduces DON level just like ZON level in corn [28, 69], while other reports deny the reduction of DON during heating [26]. On the other hand, DON level was decreased up to 35% during baking cookies. Some discrepancy of DON retention during baking bread or cakes may result from food additives, such as ammonium carbonate contained in batter [26]. During baking, isoDON, an isomer of DON was known to be formed (Figure 2). Sugita-Konishi *et al*. reported that DON level was retained 108% while cytotoxicity level was reduced slightly, but significantly after baking [53].

## 4. DON metabolites and reaction products with matrix

As described above, DON is known to decompose under some conditions and form isomers or derivatives (Figure 2), however, little is known about their toxicity. Table 5 summarizes the toxicity of DON degradation products or biological metabolites of DON. During baking, isoDON, an isomer of DON was known to be formed [51, 61]. Heating DON under alkaline conditions gave DON degradation products such as the norDON series, which shows less cytotoxicity [56]. De-epoxy DON was not only found in milk and serum in cows fed DON contaminated feed, but also found in microbial inocula from rumen fluid, soil, and contents of the large intestines of chickens (CLIC) and of swine [62, 63]. De-epoxy DON was reported to be about ten times less toxic than DON assessed by IC_50_ for inhibition of DNA synthesis in mouse 3T3 cells [20]. DON-3-glucronide, another metabolite of DON in animals, was also less toxic than DON [66]. Other metabolites of DON identified were 3-keto DON and DON-3-glucoside (DON-3G). Their biological activities remain to be elucidated.

## 5. Discussion

Wet and temperate weather occasionally causes a high occurrence of scab or FHB of wheat, which gave us dual damages. One is an agricultural loss; the reduction of wheat harvest and another is a threat for food safety; the contamination of mycotoxins in wheat grains. DON is one of the major mycotoxins in scabby wheat, of which major part is processed for foodstuffs. The study on the retention of DON during primary processing (milling) and secondary processing (cooking) has been conducted since 1980’s when scab become a major concern worldwide.

As for the selection for decontaminated kernels before processing, Seitz *et al*. [29] showed that cleaning *Fusarium*-infected wheat by a conventional commercial flow was not particularly effective in removing DON. Huff and Hagler [33] showed that density flotation could be used to separate *Fusarium*-infected wheat kernels, but the cost of drying the decontaminated grain limits commercial application. Various selection machines using air flow has been developed. Selection of *Fusarium*-infected kernels by spectrometry is underway. In all cases, the problem lies on the point that FHB level is not always correlated with the content of DON in the kernels, and the ratio of FHB to DON level varied slightly among cultivars. The correlation of DON levels with ergosterol content or ash concentration was reported, which still remains to be studied for application.

During milling process, DON is stable and not decomposed from naturally contaminated wheat. Some fractionation took place during milling and DON is more concentrated in low-grade flour streams and offals, but still present in appreciable amounts in the most refined flour streams which are intended for human consumption. These results are similar to those found in milled corn contaminated with ZON.

DON is highly water-soluble and DON level is drastically reduced during cooking using a larger quantity of boiling water if the cooking liquid is discarded, like in the case of making spaghetti. DON is very stable during thermal cooking such as baking, frying and extrusion cooking. Reduction of DON concentration is dependent on cooking time, temperature, pH, recipe, food additives, and other factors.

In summary, the available data clearly show that DON is reduced step by step during processing, but not completely removed from final products. For example, compared to the uncleaned wheat, the levels of DON were 77% in cleaned wheat, 37% in semolina, 33% in spaghetti and 20% in cooked spaghetti [44]. On the other hand, the change on the toxicity is not well studied.

Further studies should be conducted with respect to four points as follows:

1. Development of effective selection method for the discrimination of visually uninfected kernels from DON containing ones, since elimination of DON during processing is not an easy task.
2. Validation of analytical method for the assessment of the retention of DON in food production chain. There are many reports with analytical values cited in this article, but little of them describe validation data such as method recovery for each matrix, limit of detection, and intermediate precision.
3. Multiple analyses of DON, NIV and ZON, which often co-exist in scabby wheat. Gas chromatography (GC) coupled with mass spectrometry (GC-MS), high-performance liquid chromatography (HPLC), and HPLC coupled with tandem mass spectrometry (LC-MS/MS) will be applicable for the multiple analyses of *Fusarium* mycotoxins [70].
4. Elucidation of small amounts of degraded DON and conjugated DON metabolites with their toxicological effects. Toxic effects in food are not always correlated with DON content, since there are some degradation products with different toxicological effects. The chemical fate of DON during thermal cooking is also not well understood and it is unclear that reduced concentrations of DON results from decomposition of DON or from their chemical binding to compounds (sugar, protein, and others) in food matrices. Very recently, several studies have suggested that DON may occur in a bound or conjugated form. DON-3G has gained considerable interest because of its universal existence in cereals. Therefore, it might be necessary to investigate total DON in cereals including DON-3G or masked mycotoxin [71, 72]. Recent advance on the analytical instruments such as LC-MSMS and LC- time of flight (TOF)-MS will help the elucidation of small amounts of degraded DON and conjugated DON metabolites. The development of the analytical equipment combined with a practical bioassay system which can predict biological effects will greatly contribute to the toxicological study of DON in humans.

## 6. Conclusions

Research on how cleaning, milling and cooking processes affect the retention of DON in wheat have been reported from North America, Europe and Asia. DON is reduced step by step during processing, but not completely eliminated from final products. In cleaning, substantial reduction of DON is achieved by removal of FHB-infected kernels, while it has a limitation because of the discrepancy between DON content and the severity of FHB. Reduction of DON can also be performed at the stage of milling if bran and shorts (outer skin of kernels; which contains higher amount of DON) are discarded. DON is heat-stable through cooking process, while boiling in larger amount of water reduces DON content by dissolution of DON in water. In some cooking such as heating under alkaline conditions, DON is reported to degrade to DON-related chemicals whose toxicological effects remain unknown. It will be necessary to monitor the DON-related chemicals considering a mass balance of total DON as well as to assess their toxicological properties.

## Figures and Tables

**Table 1.** Toxicological effects of type B trichothecenes on animals.

Type B trichothecene	Molecular formula	Toxicity	Comments	Ref.
deoxynivalenol (DON)	C_15_H_20_O_6_	emesis and feed refusal in swine, IgA nephropathy in mice, alter humoral immunity in mice, inhibition of RNA and DNA synthesis in human cell lines, activation of MAPK in human cell lines, activation of cytokines in murine and human cells, LD_50_ in ddS strain of male mice dosed IP was 70 mg/kg; females, 76.7 mg/kg, IC_50_ for the inhibition of DNA synthesis was 1.50 mM in mouse 3T3 cells, LD_50_ values for DON were estimated to be 78 mg/kg (oral) and 49 mg/kg (i.p.) in the B6C3F1 mouse	Major mycotoxin in scabby cereals	[4, 12–21]
nivalenol (NIV)	C_15_H_20_O_7_	LD_50_ in mice dosed IP was 5 mg/kg, induced apoptosis in thymus, spleen and Peyer's patches of mice, IC_50_ for the inhibition of DNA synthesis was 1.19 mM in mouse 3T3 cells	Another major type B trichothecene co-occurred with DON	[16, 18–20, 22]
3-acetyldeoxynivalenol	C_17_H_22_O_7_	LD_50_ in ddS strain of male mice dosed IP was 49.5 mg/kg; females, 49.9 mg/kg, IC_50_ for the inhibition of DNA synthesis was 14.4 mM in mouse 3T3 cells	biosynthetic precursor of DON in fungi	[16, 20]
15-acetyldeoxynivalenol	C_17_H_22_O_7_	IC_50_ for the inhibition of DNA synthesis was 1.51 mM in mouse 3T3 cells, LD_50_ values for 15-acetylDON were 34 mg/kg (oral) and 113 mg/kg (i.p.).	biosynthetic precursor of DON in fungi	[20]
Fusarenon-X	C_17_H_22_O_8_	LD_50_ in ddS strain of male mice dosed IP was 3.3 mg/kg, IC_50_ for the inhibition of DNA synthesis was 0.72 mM in mouse 3T3 cells		[16, 20]

**Table 2.** Effects of pre-milling processes on the removal of DON.

Wheat	Mycotoxin	Treatment	Effect	Ref.
Hard Red Spring (Canada)	DON (7.1 mg/kg)	cleaning (Carter dockage tester)	DON in cleaned wheat was 4.6 mg/kg, while DON in dockage was 16.7 mg/kg	[25]
Soft White Winter(Canada)	DON	cleaning (Carter dockage tester)	cleaning did not reduce DON level	[26]
Soft wheat (USA)	DON	cleaning (combination of screening and air flow)	DON content was decreased by 16% and screening had 4.7 fold higher DON contents than cleaned wheat	[27]
wheat (USA)	DON (7.9–9.6 mg/kg)	cleaning	The percent reduction found in the cleaned wheat ranged from 6 to 19%.	[28]
Hard Red Winter (USA)	DON (0.64–5.1 mg/kg)	cleaning (Superior cylinder separators) followed by washing (Smico wheat washer)	normal, single cleaning obtained the cleaning efficiency ranging from 48% to 86% depending on DON concentration.	[29]
Canadian Western Red Spring, variety Sinton (Canada)	DON (12.5 mg/kg)	scouring	scouring reduced DON level by 22%	[30]
Canadian Western Amber Durum, variety Coulter(Canada)	DON (9.6 mg/kg)	scouring	without scouring did not affect DON level	[30]
Canadian Eastern White Winter(Canada)	DON	cleaning (Carter C-989 dockage tester) followed by fractionating (SY 300 gravity separator, Spiroll Kipp Kelly Inc.)	DON is highest in the least dense fractions	[31]
wheat naturally contaminated with DON and ZON	DON (2.4 mg/kg)	density segregation by soaking in water and 30% sucrose	removing wheat buoyant in water and then 30% sucrose decreased the DON present by 96%, and reduced the DON content by 96%	[33]
wheat naturally contaminated with DON and ZON	DON (0.6 mg/kg)	density segregation by soaking in water and 30% sucrose	removing wheat buoyant in water and then 30% sucrose decreased the DON present by 68%, and reduced the DON content by 67%	[33]

**Table 3.** DON distribution and reduction after milling.

Wheat	Mycotoxin	Treatment	Effect	Ref.
Hard Red Spring (Canada)	DON (4.6 mg/kg)	milling (Allis-Chalmers laboratory mill)	DON in 75% yield straight-grade flour was 4.1mg/kg, while 4.6, 6.9, 8.0 ppm in bran, shorts, and feed flour (red dog), respectively	[25]
Soft White Winter (Canada)	DON (0.42–0.62 mg/kg)	milling (industrially milled and pilot-milled)	milling led to a fractionation of DON, with increased levels in the outer kernel (bran, 0.98 mg/kg, for example) portions and decreased levels in the inner flour portions (break flour, 0.28 mg/kg)	[26]
soft Wheat, variety Pike, Hart, Stadler, Oasis, Pioneer, Mcnair, Arthur (USA)	DON (0.03–3.35 mg/kg)	dry milling (Miag Multomat mill)	DON was found in all fractions, which included straight-grade flour, four break flours, six reduction flours, break and reduction shorts, red dog, and bran. Mean DON concentration in straight-grade flour was about 90% of that in cleaned wheat.	[27]
wheat (USA)	DON (7.9–9.6 mg/kg)	milling (Bühler automatic laboratory mill Model MLU-202)	DON was found throughout all the milling fractions. The greatest (21 mg/kg) concentration of DON was found in the bran, and the smallest (1 mg/kg) was found in the break flour.	[28]
Hard Red Winter (USA)	DON (0.64–5.1 mg/kg)	milling (Miag Multomat Mill)	all mill fractions contained DON with lower concentration in flours and higher concentration in offals compared to cleaned wheat, DON concentration of straight-grade flour was 75% of cleaned wheat	[29]
Canadian Western Red Spring, variety Sinton (Canada)	DON (12.5 mg/kg)	milling (Bühler laboratory mill Model MLU-202)	mould counts highest in bran, 29% of DON was retained in flour	[30]
Canadian Western Amber Durum, variety Coulter (Canada)	DON (9.6 mg/kg)	milling (Bühler laboratory mill Model MLU-202)	mould counts highest in shorts, 52% of DON was retained in semolina	[30]
White Winter (Canada)		milling (Allis-Chalmers laboratory mill)		[31]
soft wheats; Red Winter (USA), White Winter (Canada)	DON (2.0 mg/kg)	dry milling	DON was distributed throughout all fractions of the milled grains; bran,red dogs and shorts: 5.2 mg/kg, straight-grade flour: 4.5 mg/kgwhile total DON recovered was 4.7 mg/kg	[35]
Hard Red Spring (Canada)	DON (7.5 mg/kg, 1.4 mg/kg)	milling by experimental mill (Allis-Chalmers mill) and commercial-scale pilot mill	no appreciable losses of DON occurred during the milling, DON concentrations raised up to two-fold in shorts and feed flour fractions	[36]
wheat (Japan)	DON (0.17mg/kg), co-contaminated with NIV and ZON	milling (Bühler experimental mill)	ca 60% of DON was remained after milling, DON content in bran was 2.7 times of that in the original wheat	[37]
wheat (Korea)	DON (0.068, 0.31 mg/kg), co-contaminated with NIV and ZON	milling (Bühler test mill)	24–48% reduction of DON in flour fractions intended for human consumption	[38]
Hard Red Spring, cultivar Glenlea, Grandin, Roblin, Taber (Canada)	DON (0.6–2.0 mg/kg)	milling (Allis-Chalmers five-stand mill)	reduction ratio of DON during milling was cultivar specific, DON level in flour was about half of wheat in Glenlea	[39]
Hard Red Winter (USA)	DON, ZON (<1.0 - >5.0 mg/kg)	milling (Bühler mill)	DON levels was highest in the bran (3.4 mg/kg) and lowest in the flour (1.5 mg/kg)	[40]
durum, cultivar Reva, DT 673, AC Melita, Durex, Hercules, DT 666, Kyle, Plenty, DT 369, Duraking (Canada)	DON (0.6–3.7 mg/kg)	milling (Allis-Chalmers five-stand mill)	about half of DON was retained in semolina of all samples	[41]
durum, cultivar Duilio, Bracco, Orobel (Italy)	DON (0.3 to 13.1 mg/kg)	milling (Bühler automatic laboratory mill Model MLU-202)	the level of DON in cleaned wheat was 77% of that in uncleaned wheat. DON levels in the screenings, bran and fine middlings were 4.1–, 1.6- and 0.6-fold, respectively, relative to the uncleaned wheat.	[44]
wheat, cultivar Seto-komugi, Asakaze-komugi (Japan)	DON, NIV (0.15–0.55 mg/kg)	milling (Bühler test mill)	DON content in bran was 2.3–2.4 times of that in the original wheat	[46]
wheat, variety Bolero (Italy)	DON (undetermine d before milling)	traditional milling with a stone mill/modern milling with a roller mill	traditional milling reduced DON level to 0.17mg/kg, while DON level became 0.36 mg/kg by modern milling	[47]
wheat (Czech Republic)	DON (0.09–2.99 mg/kg)	milling (Bühler automatic laboratory mill Model MLU-202)	the highest concentrations of DON were found in the bran, the lowest in the reduction flours. In Sulamit; uncleaned grain 108, dust 2689, cleaned grain 59, 1st-3rd break flour 42–64, 1st-3rd reduction flour 26–33, bran 117 (ng/g)	[48]
spring wheat,variety Grena, Carasso, Lana, Brusino, Toronit, Fiorina, Quarna, Nadro (Switzerland)	DON (undetermined before milling)	medium size sample mill (Bühler MLU202)	DON contents in break flour, reduction flour, shorts, bran were 16.2, 16.9, 84.4, 122.0 mg/kg respectively, in Greina variety, which showed the most severe FHB symptom	[49]

**Table 4.** Effects of secondary processing; thermal cooking.

Wheat	Mycotoxin	Treatment	Effect	Ref.
Hard Red Spring (Canada)	DON (4.1 mg/kg)	baking (30 min at 205°C)	DON was not destroyed on making bread	[25]
Soft White Winter (Canada)	DON (0.28–0.44 mg/kg)	baking cookies by a standard commercial recipe	DON level was decreased up to 35%	[26]
wheat (USA)	DON (0.52–0.31 mg/kg)	baking by an approved method of the American Association of Cereal Chemists	DON was not destroyed, but the effect on its concentration in the samples analyzed varied, the reduction ranging from 19 to 69%	[28]
Hard Red Winter (USA)	DON (0.22–5.8 mg/kg)	baking (24 min at 215 °C)	decreased DON in bread from lower DON contaminated wheat, whereas increased DON in bread from higher (>1.6 mg/kg) DON contaminated wheat	[29]
wheat (Japan)	DON (0.17 mg/kg), co-contaminated with NIV and ZON	baking (30 min at 170 °C)	baking did not affect DON content	[37]
wheat (Czech Republic)	DON (0.09–2.99 mg/kg)	baking (14 min at 210 °C)	baking had no significant effect on DON levels	[48]
	DON	baking Egyption bread (2 min at 350 °C)	DON was not reduced	[50]
Hard Red Spring, cultivar Len (USA)	DON (3.13 mg/kg)	baking (20 min at 220 °C)	L-cysteine as a food additive was significantly reduced DON (38–46%) in bread, isoDON was formed	[51]
wheat, cultivar Haruyokoi (Japan)	DON (0.71 mg/kg)	baking (35 min at 160 °C)	the level of DON was not reduced while the cytotoxicity (in Swiss mouse 3T3 fibloblasts) was reduced slightly but significantly	[53]
wheat (Argentina)	DON (150 mg/kg)	baking French bread on a pilot scale	41% of reduction of DON was observed during fermentation at 50°C	[54]
wheat (Argentina)	DON (150 mg/kg)	baking Vienna bread on a pilot scale	56% of reduction of DON was observed during fermentation at 50°C	[54]
wheat (Argentina)	DON (1.2 mg/kg)	frying (15 min at 169 °C, 2.5 min at 205 °C, 1.0 min at 243 °C)	No significant reduction of DON was observed	[55]
Canadian Western Red Spring, variety Sinton (Canada)	DON (12.5 mg/kg)	Chinese noodles (75 g of dried noodles (containing 1% NaCl and 1% Kansui, 12% moisture) were boiled in 750 mL water for 10 min and drained for 5 min	total DON retention (DON in cooked noodles and DON in cooking water solids/DON in uncooked noodles) was 57–58%, reduction of DON level was 49% after boiling	[30]
Canadian Western Red Spring, variety Sinton (Canada)	DON (12.5 mg/kg)	Japanese noodles (75 g of dried noodles (containing 1% NaCl, 12% moisture) were boiled in 750 mL water for 10 min and drained for 5 min	total DON retention (DON in cooked noodles and DON in cooking water solids/DON in uncooked noodles) was 107–115%, reduction of DON level was 40% after boiling	[30]
Canadian Western Amber Durum, variety Coulter (Canada)	DON (9.6 mg/kg)	spaghetti (75 g) made by low temperature extrusion (39°C for 28 h) was boiled in 750 mL water for 10 min and drained for 5 min	total DON retention* was 87–92%, DON concentration in cooked spaghetti made of uncooked spaghetti (DON conc. 6.2 mg/kg) was 3.5 mg/kg	[30]
Canadian Western Amber Durum, variety Coulter (Canada)	DON (9.6 mg/kg)	spaghetti (75 g) made by high temperature extrusion (70°C for 12 h) was boiled in 750 mL water for 10 min and drained for 5 min	total DON retention* was 90–92%, DON concentration in cooked spaghetti made of uncooked spaghetti (DON conc. 6.1 mg/kg) was 3.4 mg/kg	[30]
durum wheat	DON (0.3 to 13.1 mg/kg)	spaghetti (25 g, containing 1% NaCl, 11.5% moisture) was boiled in 100 mL water for 7 min and drained	total DON retention* was 88–114%, DON concentration in cooked spaghetti made of uncooked spaghetti (DON conc. 7.0 mg/kg) was 2.7 mg/kg	[44]
durum wheat	DON (0.3 to 13.1 mg/kg)	spaghetti (25 g, containing 1% NaCl, 11.5% moisture) was boiled in 125 mL water for 7 min and drained	total DON retention* was 74–89%, DON concentration in cooked spaghetti made of uncooked spaghetti (DON conc. 0.26 mg/kg) was 0.048 mg/kg	[44]
	DON (7.3 mg/kg)	the effects of sodium bisulfite, extrusion cooking under high temperature and pressure	soaking treatment with SB solution (5% SO_2_ equivalent) lowered DON to 0.8 mg/kg and to 0.3 mg/kg with following extrusion process	[52]
wheat, cultivar Hokushin (Japan)	DON (0.86 mg/kg)	Japanese noodles (50 g of dried noodles (containing 4% NaCl) were boiled in 1000 mL water for 10 min and dried at 40°C for 3 h	boiling process reduced both the level of DON and cytotoxicity (in Swiss mouse 3T3 fibloblasts)	[53]
no grain used	DON (100 mg)	cooking or baking with model heating (heat with 1 mg of model compounds: α-D-glucose as sugar model, for example)	heating under alkaline conditions decomposed DON partially and gave a mixture of norDON series	[56]
	DON	preparation of Egyptian 'balila'	preparation by using Na_2_CO_3_ solution was effective to reduce DON level	[57]
wheat	DON (1.0 mg/kg)	extrusion (140 °C –180 °C )	No reduction of DON content by simple extrusion was observed	[59]
Hard Red Spring (Canada)	DON (15.8 mg/kg)	superheated steam	48% of reduction of DON was occurred at 185 °C in the processing times of 6 min	[60]

*total DON retention: the value of DON in cooked spaghetti and in cooking water solids / the value of DON in uncooked spaghetti

**Table 5.** Toxicological effects of DON derivatives on animals.

Compound	Molecular formula	Toxicity	Comments	Ref.
3-keto DON (a)*	C_15_H_18_O_6_	no data available	transformed product of DON under aerobic conditions by microbial cultures	[64, 65]
isoDON (b)	C_15_H_20_O_6_	no data available	found in bread	[51, 61]
de-epoxy DON (c)	C_15_H_20_O_5_	IC_50_ for inhibition of DNA synthesis was 83.0 mM in mouse 3T3 cells	found in milk and serum in cows fed DON contaminated feed	[20, 62, 63]
norDON A (d)	C_14_H_18_O_5_	less cytotoxic up to 100 mM in human kidney epithelial IHKE cells compared to DON (EC_50_=1.1 μm)	detected in 29–66% of the samples in mean concentrations ranging from 3 to 15 mg/kg during heating under alkaline conditions	[56]
norDON B (e)	C_14_H_18_O_5_	less cytotoxic up to 100 mM in human kidney epithelial IHKE cells compared to DON (EC_50_=1.1 μm)	detected in 29–66% of the samples in mean concentrations ranging from 3 to 15 mg/kg during heating under alkaline conditions	[56]
norDON C (f)	C_14_H_18_O_5_	less cytotoxic up to 100 mM in human kidney epithelial IHKE cells compared to DON (EC_50_=1.1 μm)	detected in 29–66% of the samples in mean concentrations ranging from 3 to 15 mg/kg during heating under alkaline conditions	[56]
DON-3-glucuronide (g)	C_21_H_28_O_12_	no toxicity at up to 270 mM in human K562 erythroleukemia cell line, and significant difference in immunotoxicity compared to DON	detoxification product of DON in plasma or urine	[66]
DON-3-glucoside (DON-3G) (h)	C_21_H_30_O_11_	no data available	in almost all investigated DON contaminated cereals also contain DON-3G	[67, 68]

## Abbreviations:

DON: deoxynivalenol
FHB: *Fusarium* head blight
ZON: zearalenone
NIV: nivalenol
DON-3G: DON-3-glucoside

## References

[1] Creppy EE (2002). Update of survey, regulation and toxic effects of mycotoxins in Europe. Toxicol. Lett.

[2] McLean M (1996). The phytotoxicity of Fusarium metabolites: An update since 1989. Mycopathologia.

[3] Seitz LM, Bechtel DB (1985). Chemical, physical and microscopical studies of scab-infected hard red winter wheat. J. Agric. Food Chem.

[4] Gutleb AC, Morrison E, Murk AJ (2002). Cytotoxicity assays for mycotoxins produced by Fusarium strains: a review. Environ. Toxicol. Pharmacol.

[5] Lee US, Jang HS, Tanaka T, Toyasaki N, Sugiura Y, Oh YJ, Cho CM, Ueno Y (1986). Mycological survey of Korean cereals and production of mycotoxins by *Fusarium* isolates. Appl. Environ. Microbiol.

[6] Osborne LE, Stein JM (2007). Epidemiology of *Fusarium* head blight on small-grain cereals. Int. J. Food Microbiol.

[7] Mirocha CJ, Pathre SV, Schauerhamer B, Christensen CM (1976). Natural occurrence of *Fusarium* toxins in feedstuff. Appl. Environ. Microbiol.

[8] Morgavi DP, Riley RT (2007). An historical overview of field disease outbreaks known or suspected to be caused by consumption of feeds contaminated with *Fusarium* toxins. Anim. Feed Sci. Technol.

[9] Ueno Y, Ishii K, Sato N, Ohtsubo K (1974). Toxicological approaches to the metabolites of Fusaria. VI. Vomiting factor from moldy corn infected with *Fusarium* spp. Jpn. J. Exp. Med.

[10] Desjardins AE, Hohn TM, McCormick SP (1993). Trichothecenes biosynthesis in Fusarium species: Chemistry, genetics, and significance. Microbiol. Rev.

[11] Ehrlich K, Daigle KW (1987). Protein synthesis inhibition by 8-oxo-12,13-epoxytrichothecenes. *Biochim. Biophys.*. Acta.

[12] Rotter BA, Prelusky DB, Pestka JJ (1996). Toxicology of deoxynivalenol (Vomitoxin). J. Toxicol. Environ. Health.

[13] Dong W, Pestka JJ (1993). Persistent dysregulation of IgA production and IgA nephropathy in the B6C3F1 mouse following withdrawal of dietary vomitoxin (deoxynivalenol). Fundam. Appl. Toxicol.

[14] Tryphonas H, Iverson F, So Y, Nera EA, Mcguire PF, O'Grady L, Clayson DB, Scott PM (1986). Effects of deoxynivalenol (vomitoxin) on the humoral and cellular immunity of mice. Toxicol. Lett.

[15] Pestka JJ, Bondy GS (1990). Alteration of immune function following dietary mycotoxin exposure. Can. J. Physiol. Pharmacol.

[16] Cole RJ, Cox RH (1981). Handbook of toxic fungal metabolites;.

[17] Forsell JH, Jensen R, Tai J-H, Witt M, Lin WS, Pestka JJ (1987). Comparison of acute toxicities of deoxynivalenol (vomitoxin) and 15-acetyldeoxynivalenol in the B6C3F1 mouse. Food Chem. Toxicol.

[18] Rocha O, Ansari K, Doohan FM (2005). Effects of trichothecene mycotoxins on eukaryotic cells. Food Addit. Contam.

[19] Pestka JJ, Smolinski AT (2005). Deoxynivalenol: toxicology and potential effects on humans. J. Toxicol. Environ. Health B Crit. Rev.

[20] Sundstøl Eriksen G, Pettersson H, Lundh T (2004). Comparative cytotoxicity of deoxynivalenol, nivalenol, their acetylated derivatives and de-epoxy metabolites. Food Chem. Toxicol.

[21] Canady RA, Coker RD, Egan SK, Krska R, Kuiper-Goodman T, Olsen M, Pestka J, Resnik S, Schlatter J (2001). Deoxynivalenol. WHO/IPCS safety evaluation of certain mycotoxins in food. WHO Food Addit. Ser.

[22] Poapolathep A, Ohtsuka R, Kiatipattanasakul W, Ishigami N, Nakayama H, Doi K (2002). Nivalenol-induced apoptosis in thymus, spleen and Peyer's patches of mice. Exp. Toxicol. Pathol.

[23] European Commission, Scientific Committee on Food Opinion of the Scientific Committee on Food on Fusarium toxins, Part 6: Group evaluation of T-2 toxin, HT-2 toxin, nivalenol and deoxynivalenol 2002; http://ec.europa.eu/food/fs/sc/scf/out123_en.pdf

[24] Yuen GY, Schoneweis SD (2007). Strategies for managing *Fusarium* head blight and deoxynivalenol accumulation in wheat. Int. J. Food Microbiol.

[25] Scott PM, Kanhere SR, Lau P-Y, Dexter JE, Greenhalgh R (1983). Effects of Experimental flour milling and breadbaking on retention of deoxynivalenol (vomitoxin) in hard red spring wheat. Cereal Chem.

[26] Young JC, Fulcher RG, Hayhoe JH, Scott PM, Dexter JE (1984). Effect of milling and baking on deoxynivalenol (vomitoxin) content of eastern Canadian wheats.. J. Agric. Food Chem..

[27] Seitz LM, Yamazaki WT, Clements RL, Mohr HE, Andrews L (1985). Distribution of deoxynivalenol in soft wheat mill streams. Cereal Chem.

[28] Abbas HK, Mirocha CJ, Pawlosky RJ, Pusch DJ (1985). Effect of cleaning, milling, and baking on deoxynivalenol in wheat. Appl. Environ. Microbiol.

[29] Seitz LM, Eustace WD, Mohr HE, Shogren MD, Yamazaki WT (1986). Cleaning, milling and baking tests with hard red winter wheat containing deoxynivalenol. Cereal Chem.

[30] Nowicki TW, Gaba DG, Dexter JE, Matsuo RR, Clear RM (1988). Retention of the *Fusarium* mycotoxin deoxynivalenol in wheat during processing and cooking of spaghetti and noodles. J. Cereal Sci.

[31] Tkachuk R, Dexter JE, Tipples KH, Nowicki TW (1991). Removal by specific gravity table of tombstone kernels and associated trichothecene from wheat infected with *Fusarium* head blight. Cereal Chem.

[32] Trigo-Stockli DM (2002). Effect of processing on deoxynivalenol and other trichothecenes. Adv. Exp. Med. Biol.

[33] Huff WE, Hagler WM (1985). Density segregation of corn and wheat naturally contaminated with aflatoxin, deoxynivalenol and zearalenone. J. Food Prot.

[34] Kos G, Lohninger H, Krska R (2003). Development of a method for the determination of *Fusarium* fungi on corn using mid-infrared spectroscopy with attenuated total reflection and chemometrics. Anal. Chem.

[35] Hart LP, Braselton WE (1983). Distribution of vomitoxin in dry milled fractions of wheat infected with *Gibberella zeae*. J. Agric. Food Chem.

[36] Scott PM, Kanhere SR, Dexter JE, Brennan PW, Trenholm HL (1984). Distribution of the trichothecene mycotoxin deoxynivalenol (vomitoxin) during the milling of naturally contaminated hard red spring wheat and its fate in baked products. Food Addit. Contam.

[37] Tanaka T, Hasegawa A, Yamamoto S, Matsuki Y, Ueno Y (1986). Residues of *Fusarium* mycotoxins, nivalenol, deoxynivalenol and zearalenone, in wheat and processed food after milling and baking. J. Food Hyg. Soc. Japan.

[38] Lee U-S, Jang H-S, Tanaka T, Oh Y-J, Cho C-M, Ueno Y (1987). Effect of milling on decontamination of *Fusarium* mycotoxins nivalenol, deoxynivalenol, and zearalenone in Korean wheat. J. Agric. Food Chem.

[39] Dexter JE, Clear RM, Preston KR (1996). *Fusarium* head blight: effect on the milling and baking of some Canadian wheats. Cereal Chem.

[40] Trigo-Stockli DM, Deyoe CW, Satumbaga RF, Pedersen JR (1996). Distribution of deoxynivalenol and zearalenone in milled fractions of wheat. Cereal Chem.

[41] Dexter JE, Marchylo BA, Clear RM, Clarke JM (1997). Effect of *Fusarium* head blight on semolina milling and pasta-making quality of durum wheat. Cereal Chem.

[42] Hart LP, Casper H, Schabenberger O, Ng P (1998). Comparison of gas chromatography-electron capture and enzyme-linked immunosorbent assay for deoxynivalenol in milled fractions of naturally contaminated wheat. J. Food Prot.

[43] Manthey FA, Wolf-Hall CE, Yalla S, Vijayakumar C, Carlson D (2004). Microbial loads, mycotoxins, and quality of durum wheat from the 2001 harvest of the northern plains region of the United States. J. Food Prot.

[44] Visconti A, Haidukowski EM, Pascale M, Silvestri M (2004). Reduction of deoxynivalenol during durum wheat processing and spaghetti cooking. Toxicol. Lett.

[45] Hazel CM, Patel S (2004). Influence of processing on trichothecene levels. Toxicol. Lett.

[46] Tanaka K, Hara N, Goto T, Manabe M (1999). Reduction of mycotoxins contamination by processing grain. Proc. Int. Symp. Mycotoxicol.

[47] Palpacelli V, Beco L, Ciani M (2007). Vomitoxin and zearalenone content of soft wheat flour milled by different methods. J. Food Prot.

[48] Lancova K, Hajslova J, Kostelanska M, Kohoutkova J, Nedelnik J, Moravcova H, Vanova M (2008). Fate of trichothecene mycotoxins during the processing: milling and baking. Food Addit. Contam.

[49] Gärtner BH, Munich M, Kleijer G, Mascher F (2008). Characterisation of kernel resistance against Fusarium infection in spring wheat by baking quality and mycotoxin assessments. Eur. J. Plant Pathol.

[50] El-Banna AA, Lau P-Y, Scott PM (1983). Fate of mycotoxins during processing of foodstuffs II-Deoxynivalenol (vomitoxin) during making of Egyptian bread. J. Food Prot.

[51] Boyacioglu D, Hettiarachchy NS, D’appolonia BL (1993). Additives affect deoxynivalenol (vomitoxin) flour during breadbaking. J. Food Sci.

[52] Accerbi M, Rinaldi VE, Ng PK (1999). Utilization of highly deoxynivalenol-contaminated wheat via extrusion processing. J. Food Prot.

[53] Sugita-Konishi Y, Park BJ, Kobayashi-Hattori K, Tanaka T, Chonan T, Yoshikawa K, Kumagai S (2006). Effect of cooking process on the deoxynivalenol content and its subsequent cytotoxicity in wheat products. Biosci. Biotechnol. Biochem.

[54] Samar MM, Neira MS, Resnik SL, Pacin A (2001). Effect of fermentation on naturally occurring deoxynivalenol (DON) in Argentinean bread processing technology. Food Addit. Contam.

[55] Samar MM, Resnik SL, Gonzalez HHL, Pacin AM, Castillo MD (2007). Deoxynivalenol reduction during the frying process of turnover pie coveres. Food Control.

[56] Bretz M, Beyer M, Cramer B, Knecht A, Humpf HU (2006). Thermal degradation of the *Fusarium* mycotoxin deoxynivalenol. J. Agric. Food Chem.

[57] Ragab WS, Drusch S, Schnieder F, Beyer M (2007). Fate of deoxynivalenol in contaminated wheat grain during preparation of Egyptian 'balila'. Int. J. Food Sci. Nutr.

[58] Castells M, Marin S, Sanchis V, Ramos AJ (2005). Fate of mycotoxins in cereals during extrusion cooking: A review. Food Addit. Contam.

[59] Scudamore KA, Guy RC, Kelleher B, MacDonald SJ (2008). Fate of the fusarium mycotoxins, deoxynivalenol, nivalenol and zearalenone, during extrusion of wholemeal wheat grain. Food Addit. Contam.

[60] Cenkowski S, Pronyk C, Zmidzinska D, Muir WE (2007). Decontamination of food products with superheated steam. J. Food Eng.

[61] Greenhalgh R, Gilbert J, King RR, Blackwell BA, Startin JR, Shepherd MJ (1984). Synthesis, characterization, and occurrence in bread and cereal products of an isomer of 4-deoxynivalenol (vomitoxin). J. Agric. Food Chem.

[62] Yoshizawa T, Cote LM, Swanson SP, Buck WB (1986). Confirmation of DOM-1, a de-epoxidation metabolite of deoxynivalenol, in biological fluids of lactating cows. Agric. Biol. Chem.

[63] He P, Young LG, Forsberg C (1992). Microbial transformation of deoxynivalenol (vomitoxin). Appl. Environ. Microbiol.

[64] Völkl A, Vogler B, Schollenberger M, Karlovsky P (2004). Microbial detoxification of mycotoxin deoxynivalenol. J. Basic Microbiol.

[65] Shima J, Takase S, Takahashi Y, Iwai Y, Fujimoto H, Yamazaki M, Ochi K (1997). Novel detoxification of the trichothecene mycotoxin deoxynivalenol by a soil bacterium isolated by enrichment culture. Appl. Environ. Microbiol.

[66] Wu X, Murphy P, Cunnick J, Hendrich S (2007). Synthesis and characterization of deoxynivalenol glucuronide: Its comparative immunotoxicity with deoxynivalenol. Food Chem. Toxicol.

[67] Sewald N, Lepschy von Gleissenthall J, Schuster M, Müller G, Aplin RT (1992). Structure elucidation of a plant metabolite of 4-desoxynivalenol. Tetrahedron Asymmetr.

[68] Berthiller F, Dall'Asta C, Schuhmacher R, Lemmens M, Adam G, Krska R (2005). Masked mycotoxins: determination of a deoxynivalenol glucoside in artificially and naturally contaminated wheat by liquid chromatography-tandem mass spectrometry. J. Agric. Food Chem.

[69] Abbas HK, Mirocha CJ, Rosiles R, Carvajal M (1988). Decomposition of zearalenone and deoxynivalenol in the process of making tortillas from corn. Cereal Chem.

[70] Kushiro M, Tanaka K, Miyazaki S, Nagata T (2006). Advances of liquid chromatographic determination of fumonisins; potential mycotoxins for humans. Curr. Pharm. Anal.

[71] Liu Y, Walker F, Hoeglinger B, Buchenauer H (2005). Solvolysis procedures for the determination of bound residues of the mycotoxin deoxynivalenol in fusarium species infected grain of two winter wheat cultivars preinfected with barley yellow dwarf virus. J. Agric. Food Chem.

[72] Zhou B, Li Y, Gillespie J, He GQ, Horsley R, Schwarz P (2007). Doehlert matrix design for optimization of the determination of bound deoxynivalenol in barley grain with trifluoroacetic acid (TFA). J. Agric. Food Chem.

